# Visual motion sensitivity and driving performance and safety

**DOI:** 10.3758/s13423-025-02775-6

**Published:** 2026-03-09

**Authors:** Joanne M. Wood, Alex A. Black, Philippe F. Lacherez, Allison M. McKendrick

**Affiliations:** 1 Centre for Vision and Eye Research, Optometry and Vision Science, Queensland University of Technology, Victoria Park Road, Kelvin Grove, Brisbane, QLD 4059 Australia; 2 School of Psychology, QUT, Brisbane, QLD Australia; 3 Discipline of Optometry, School of Allied Health, University of Western Australia, Perth, WA Australia; 4 Lions Eye Institute, Perth, WA Australia; 5 Department of Optometry and Vision Sciences, The University of Melbourne, Melbourne, VIC Australia

**Keywords:** Motion sensitivity, Driving performance, Motor vehicle crash risk, Driving safety, Minimum displacement thresholds, Drift rate, Optic flow, Ageing, Vision impairment

## Abstract

Perceiving and interpreting motion in the visual world is an important and complex visual process involved in activities such as driving, which involves the motion of both the driver’s own vehicle and that of other road users. Research has explored the association between tests of motion sensitivity and a range of indices of driving performance and safety, to better understand the role of motion sensitivity in driving and its ability to predict driving performance and safety. This review provides an overview of research that has explored associations between motion sensitivity tests and measures of driving performance and safety. Collectively, the findings suggest that motion sensitivity is important in the timely detection of hazards, as well as for visually guided vehicle control behaviours (e.g., lane-keeping). Impaired motion sensitivity has been shown to be associated with delayed hazard response times in computer-based tests, impaired driving performance assessed in closed and open road studies, as well as increased crash risk. Given the many driving performance outcomes in on-road and simulator studies related to motion sensitivity, further work should explore motion sensitivity as a risk factor for unsafe driving performance and collision involvement in older adults.

## Introduction

Interpreting motion in the visual world is a critical but complex visual process for activities such as driving, which involves the motion of both the driver’s vehicle and that of other road users. Accordingly, studies have explored the association between measures of motion sensitivity and indices of driving performance and safety, with the aim of better understanding the role of motion perception in driving and identifying its predictive capacity for driving.

In this review, we broadly define ‘motion sensitivity’ as the ability of a person to identify or interpret motion in the visual environment. We first introduce and overview key concepts and mechanisms of human visual motion sensitivity relevant to driving, followed by an overview of research that has explored associations between tests of motion sensitivity and measures of driving performance and safety.

This review represents a contemporary synthesis of current literature on motion sensitivity and driving. While we predominantly present a narrative review, the core literature on motion and driving was searched using MeSH terms and keywords that incorporated the following key concepts: drivers of automobiles, visual motion sensitivity, and motor vehicle crash risk and driving performance. The search was conducted within five electronic databases (PUBMED, EMBASE, PsycINFO, Medline (via EBSCOhost), and TRID using a date range of 1980–2024. Studies that were not reported in English, were not full text, or were reviews rather than primary research were excluded.

### Motion perception and relevance to driving

**Fig. 1:**
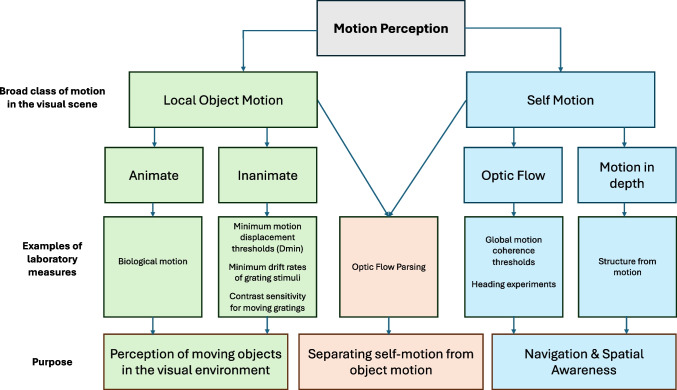
A taxonomy of human motion perception, with emphasis on attributes of relevance to driving

This section discusses the neural underpinnings and human behavioural aspects of several commonly studied aspects of motion sensitivity that are considered potentially relevant for driving, along with a description of the methods used to measure these aspects of motion sensitivity. It should be noted that there are many other reviews of visual motion processing from both a neurobiological and a psychological perspective (e.g., Nishida, 2011; Nishida et al., 2018). The aim here is not to replicate these reviews, but rather to introduce readers to aspects of motion sensitivity that may have a specific role in the task of driving. As such, this is not an exhaustive review of motion perception. The rationale for inclusion of specific motion sensitivity tasks was based on evidence of a relationship with some aspect of driving behaviour or if the consensus from the literature is that the task measures aspects of motion perception that are highly likely to be relevant to driving. Figure 1 provides a taxonomy of the tests of motion perception included in this review.

The simplest aspects of motion encoding include the ability to detect small motion displacements, necessary to detect and avoid hazards in the visual scene, such as pedestrians or cyclists moving relative to their background. Motion displacement thresholds describe the smallest detectable displacement of a target between one frame and the next within a movie sequence. This minimum displacement threshold is often referred to as D_min_. The visual system has exquisite sensitivity to detect these tiny local motion changes, with thresholds being of the order of 10 sec of arc under optimal settings (Legge & Campbell, 1981). Because more complex motion tasks are dependent on the ability to detect these motion displacements, threshold motion tasks have been commonly used as an index of motion sensitivity in studies interested in relating motion sensitivity to driving (see later sections). There are a variety of methods available for measuring D_min_ thresholds, including simple line stimuli (Legge & Campbell, 1981; Tyler & Torres, 1972) or extended field random-dot-kinematograms (RDK) (Bullimore et al., 1993).

An alternative method to measure minimum perceptible motion displacements is to determine the threshold velocity for detection of motion of a grating stimulus. The task measures how slowly a grating can drift, and the direction of motion detected (Lacherez, Au et al., 2014a). Grating stimuli permit thresholds to be determined for a variety of spatial frequencies, so can provide insight into spatial and temporal tuning of the neural encoding of motion, and have been widely used for the measurement of contrast thresholds for moving stimuli (e.g., Venkataraman et al., 2017). Contrast thresholds can be measured for either motion detection (lowest contrast to detect the presence of a moving object) or motion discrimination (lowest contrast to discriminate the direction of a moving object). Discrimination involves comparing a motion feature (e.g., velocity or direction) between a target stimulus and a reference. In the velocity discrimination case (Cropper, 1994; McKee, 1981) the observer is required to identify the fastest of two gratings (which in the limiting case is when the reference is stationary, and the task becomes a motion detection threshold).

The spatial and temporal tuning of human motion detection and discrimination for grating stimuli has been correlated with neuronal tuning properties of directionally selective neurons in early stages of the visual system, such as primary visual cortex (V1) (Aaen-Stockdale & Bowns, 2006), hence human performance on these tasks can be affected by diseases that impact on the earlier stages of visual processing (eye through to primary visual cortex).

Of potential relevance to detection and avoidance of hazards in the visual scene is the threshold duration required to reliably interpret the direction of motion of an object. Note, this is not a reaction time, but instead the duration that the stimulus needs to be presented for the observer to discriminate its motion direction. Duration thresholds are typically measured psychophysically using suprathreshold stimuli, such as gratings, and are highly dependent on the contrast, size, and drift rate of the stimulus (Borghuis et al., 2019; Tadin et al., 2003). Despite being seemingly counterintuitive, for a given drift speed, larger, high-contrast stimuli require a longer stimulus duration for correct identification of motion direction than smaller, low-contrast, stimuli (Tadin et al., 2003). This phenomenon is considered a perceptual analogue of neuronal centre-surround suppression, and shows similar spatial scaling to centre-surround neurons in cortical area V5 (middle temporal areas, MT) (Tadin et al., 2003), which is a motion-processing area widely connected to other areas of the cortex considered important for the processing of complex motion fields (Wild & Treue, 2021).

Because the entire visual scene is moving when we move through the world, the visual system also makes use of wide-field motion cues, and these are often noisy or ambiguous. Motion sensitivity tests that explore this wide-field integrative capacity typically use stimuli constructed of RDKs and allow manipulation of the level of signal to noise in the motion stimulus. A widely used task is the ‘global motion coherence’ threshold task, which is typically measured with RDKs where a subset of dots moves in a common direction within a field of randomly moving dots (Edwards & Badcock, 1994). The proportion of dots moving in a common direction is varied relative to the proportion of noise dots to determine a coherence threshold for correct identification of motion direction. For this task to assess global motion integration, the moving dots need to be selected randomly on each frame of the movie sequence so that motion direction cannot be determined by tracking the local motion of individual dots.

The earliest stage of visual processing in the brain that has the neuronal architecture capable of interpreting global motion cues is area V5/MT (Newsome & Pare, 1988). Consequently, difficulty interpreting global motion cues can arise due to reduced input from earlier stages of visual processing (e.g., damage to the eye or primary visual cortex) or may arise from specific abnormalities within the extra striate visual motion-processing areas; however, it is worth noting here that the pathway from V1 is not the only input to V5/MT, which also receives input from the superior colliculus via the pulvinar (Berman & Wurtz, 2008).

When driving, self-motion of the driver creates wide-field optic flow which provides information about the driver’s direction of motion (heading) (van den Berg, 1992). Neurons with wide-field architecture that respond to optic flow type stimuli are present in area MST, which receives direct input from area V5/MT (Wild & Treue, 2021). A process known as flow parsing distinguishes self-motion from real motion of other objects in the visual scene (Warren & Rushton, 2007, 2009). There is also increasing evidence that cortical areas V3A and V6 process visual egomotion and are important for flow parsing (Galletti & Fattori, 2003; Pitzalis et al., 2006; Prabhakar et al., 2024). Selective damage to these motion-specific areas (e.g., due to localised stroke) can result in motion-processing deficits yet spare typical clinical measures of vision that are regularly used for driving assessments such as visual acuity (see, e.g., Prabhakar et al., 2024). In addition to flow parsing, motion parallax also provides key information about depth. A recent review details current understanding of how the human brain computes motion and depth from self-motion (Xu & DeAngelis, 2025).

Flow parsing enables processing of the relative distance of various moving objects in three-dimensional space considering the observer’s egomotion. While driving, the driver specifically needs to identify hazardous moving objects from the moving background. Dependent on object characteristics and ambient light levels, there may be many cues to assist with segmentation of hazards, including colour and contrast of the object; however, when these cues are not available, the visual system is able to segment objects using motion information alone. Specifically, a different direction or speed of motion of the object relative to its background enables segmentation. Psychophysically, this can be explored using motion-discrimination tasks where the object is embedded within a moving background. A specific case is ‘motion-defined form’, where the object is not apparent in the scene unless it moves, such as when the object and the background are constructed from identical random dot textures (Norman et al., 2020; Tadin et al., 2019). In this case, when the object moves at a different speed or direction to the background, it becomes visible. There are different aspects of motion-defined form that can be measured, such as the threshold motion velocity difference for detection or the stimulus duration threshold (minimum duration that the stimulus needs to be present for correct identification of a moving object from its moving background). Individual differences in motion segmentation ability, including those arising from older age, have been directly linked to centre-surround motion suppression strength (such as that measured with threshold duration exposure for high-contrast large stimuli) (Tadin et al., 2019), supporting an ecological role for antagonistic centre-surround motion suppression in the ability to segment objects in the visual scene. In addition to two-dimensional (2D) motion, motion-defined form can also enable three-dimensional (3D) structures of objects to be interpreted from their pattern of motion (often referred to as ‘structure from motion’ or ‘kinetic depth effect’); in this case, random dot stimuli contain velocity cues consistent with an object rotating in space (Treue et al., 1991).

In summary, motion processing is highly complex, beginning with the ability of the visual system to resolve minimal motion cues, such as motion detection and discrimination of motion direction. Many salient targets in the driving scene (e.g., vehicles, buses) are likely to be moving at a supra-threshold speed, with moving objects of interest (e.g., other vehicles, pedestrians) requiring segmentation from the moving visual scene in as short a time as possible. This task relies on the temporal fidelity of the retina but also necessitates suppression of irrelevant wide-field background motion. At the same time, wide-field optic flow assists with correctly perceiving the speed and direction of self-motion, and flow parsing enables this self-motion to be differentiated from the motion of externally moving objects in the visual scene. Drivers are required to correctly perceive the potential time to collision of a hazard and to correctly perceive gaps in traffic, as well as peripheral information important for lane-keeping, speed control, and detecting potential hazards in the periphery, prompting central fixation (Crundall et al., 2002; Summala et al., 1996). The requirements for the visual system to interpret motion signals vary as a function of light levels, which may be relevant to night-time driving, where light levels are often low, objects may be of low contrast, and there are changing light levels dependent on road lighting and oncoming vehicles. Given this highly complex series of requirements, it is not surprising that the relationship between specific laboratory assessments of motion sensitivity and driving performance is an area of active research and debate and is the focus of the remainder of this review.

### Studies of motion sensitivity and driving

**Fig. 2:**
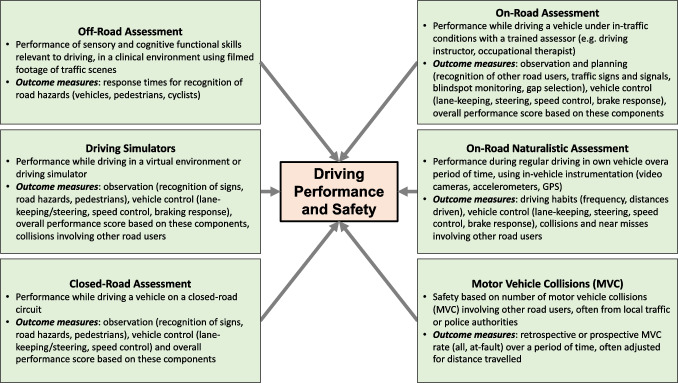
Framework of methodologies/metrics of driving performance and safety included in studies of motion perception and driving discussed in this review. More detailed descriptions of these metrics are provided in Owsley et al. (2015) and Wood (2022)

The association between motion sensitivity and driving performance and safety has been explored in a range of studies. The tests used to assess motion sensitivity have varied, and as discussed earlier in this review, assess different aspects of motion sensitivity (likely processed at different levels of the visual pathways and in other cortical areas). The terms performance and driving safety as outcome measures are used broadly in the vision and driving literature, and can refer to a range of different measures including self-reported driving performance and self-reported crash risk, laboratory-based assessments, driving performance assessed in a range of settings, and driving safety, as derived from various types of crash data. These measures reflect different aspects of driving and its component skills; however, they often make interpretation of the vision and driving literature confusing, given that they measure driving from different perspectives (Owsley et al., 2015). As this review focuses on the association between motion sensitivity and a range of different driving measures, it is useful to briefly describe the differences between these outcomes. Note that we have not included self-reported measures of driving in this review, given that they do not necessarily reflect actual driving performance (Wood, Lacherez et al., 2013b) or state-recorded crash risk (McGwin et al., 1998). In addition, we have only included laboratory-based measures of driving performance that incorporate moving presentations of driving scenes that provide cues that are likely to be associated with driving performance and safety outcomes. A framework of the methods used to assess driving performance and safety described in the next section is presented in Fig. 2.

#### Driving outcome measures

*Laboratory-based* indices of driving performance and safety have been widely used in studies of vision and driving; however, in this review we refer only to the Hazard Perception Test (HPT), given it is the only one of these commonly used tests that includes motion cues; off-road tests of driving performance that do not incorporate motion cues, such as the UFOV (Useful Field of View), Drive Safe, and Drive Aware are not included. The HPT assesses the ability to detect objects and situations that might represent a hazard in videos of traffic scenes presented under laboratory-based conditions (Crabb et al., 2010; Horswill et al., 2008; Lee et al., 2016; Underwood et al., 2005; Wetton et al., 2011). Videos are presented via computer-based software and comprise recordings of real traffic situations containing a range of traffic hazards, filmed from the driver’s perspective. Participants respond as quickly as possible when they detect the appearance of a traffic hazard by clicking the computer mouse or pressing the touch screen on the location of the hazard. Delayed HPT response times are associated with crash involvement in both retrospective and prospective studies (Darby et al., 2009; Horswill et al., 2015; McKenna & Horswill, 1999), as well as with increased heavy braking events during real-world driving (Hill et al., 2019).

*Driving performance* refers to the quality of driving based on vehicle kinematics and hazard avoidance. Driving performance measures include aspects of vehicle control (e.g., steering, lane-keeping, speed control) and hazard avoidance, which involves road observation and hazard detection (e.g., appropriate scanning of the road, anticipation of hazardous situations, detection of pedestrians) which are recorded using in-vehicle instrumentation that includes GPS, accelerometers, and video-based measures of the road ahead as well as the participant’s responses to the road environment. A number of studies of motion sensitivity and driving have included measures of driving performance undertaken on closed road circuits, as well as when driving on the open road under in-traffic conditions, and have used cross-sectional designs (e.g., Wood, Black, Dingle et al., 2021b), where visual motion sensitivity and driving are measured at the same time point and the association between these measures is determined.

*Driving safety* is typically defined by the number of motor vehicle collisions (MVCs), obtained from state or police authorities or through naturalistic driving studies, and expressed as the rate of MVCs over a given period, or as a function of distance driven. At-fault MVCs are considered most useful in understanding the role of vision in driving, with stronger associations with vision impairment when at-fault, rather than when all crashes are considered (Cross et al., 2009). However, while MVCs are the gold standard in terms of driving safety, limitations are that not all collisions are reported to authorities, and importantly, they do not provide insight into the mechanisms underlying the impact of vision impairment on driving safety. For example, a specific type of vision impairment may be associated with increased MVC risk, but crash data do not provide insight regarding why the crash occurred. Conversely, naturalistic driving provides video footage of the driving behaviours that occurred immediately prior to an MVC (which might include poor lane-keeping or poor gap judgements), as well as details of the MVC and who is likely to have been at fault.

The next section includes studies that have reported on associations between laboratory-based tests of motion sensitivity and driving outcome measures.

#### Motion sensitivity measures and driving

Tests that quantify the smallest amount of motion needed to accurately determine the direction of movement have been included in many driving studies. Studies have included high-resolution (high spatial frequency) random dot patterns, to assess *motion displacement thresholds*, as well as low-resolution (low spatial frequency) drifting gratings to assess *minimum drift rates* or *motion contrast thresholds,* and are discussed in the following sections.

*Motion displacement thresholds* are likely to be relevant to the driver in terms of detecting the movement of other road users within the driving scene, and are typically measured using RDKs*,* with D_min_ describing the minimum motion-displacement threshold. Numerous studies have explored the association between *central* D_min_ and a range of outcome measures including HPT response times and driving performance measured under both closed and open road conditions.

Impairments in D_min_ were significantly (*p* < 0.05) associated with delayed HPT response times in two separate studies of visually normal participants of a range of ages (Lacherez, Au et al., 2014a; Lacherez, Turner et al., 2014b). A more recent study of visually normal drivers of a range of ages also showed that impaired D_min_ was significantly associated with delayed HPT times even when adjusted for age (*p* = 0.02) (Sepulveda et al., 2023). The association between D_min_ and HPT times has also been reported in those with vision impairment associated with ocular disease. In older drivers with glaucoma and visually normal controls, impaired D_min_ was associated with delayed HPT response times in age-adjusted analyses (*p* = 0.008) (Lee et al., 2018). These findings were supported by a larger study of older drivers that included those with vision impairment associated with ocular diseases including cataract, age-related macular degeneration and glaucoma, and visually normal controls. Findings demonstrated that impaired D_min_ thresholds were significantly associated with delayed HPT responses (*p* *<* 0.001), which remained significant even when adjusted for measures of visual acuity and visual fields (Wood, Black, Anstey et al., 2021a). The role of motion sensitivity and visual acuity on HPT responses was also explored in young, visually normal participants, whose HPT detection rates and response times were assessed in the absence and presence of an inter-frame mask that interrupted motion cues, as well as with or without a diffusing filter that simulated low visual acuity (20/120 on average) (Moharrer et al., 2020). When motion was disrupted, there was a significant (*p* = 0.016) reduction in a combined performance score (that included response times and detection rates); however, the diffuser did not significantly impact the scores (p = 0.42), suggesting that motion perception has a more important role than visual acuity for hazard perception.

Only one study has explored the association between measures of both *central* and *peripheral* motion sensitivity, including motion contrast, translational global motion, biological motion, and D_min_ and HPT response times. While *central* motion contrast and D_min_ thresholds were both significantly (*p* = 0.02) associated with HPT response times, only *peripheral* D_min_ (15º eccentricity) was significantly (*p* = 0.05) associated with HPT response times in age-adjusted analyses, while the associations between the other peripheral motion tests (motion contrast, translational global motion, biological motion) and HPT response times were not significant_._ This finding suggests that in visually normal adults, assessment of *peripheral* motion sensitivity does not provide additional information regarding hazard response times to that of *central* motion sensitivity (Sepulveda et al., 2023); however, it remains unclear whether *peripheral* motion tests may be more relevant in those individuals with peripheral vision loss such as glaucomatous field defects.

The association between *central* D_min_ and driving performance has also been explored in a series of closed and open road studies. Impaired D_min_ performance was the most significant predictor of reduced overall driving scores as measured in daytime closed road studies of a group of licensed drivers (young, middle-aged and older participants with normal vision, and older participants with a range of ocular diseases), and was retained in the overall predictive model of driving scores (Wood, 2002). More recently, impaired D_min_ performance was also significantly associated with overall closed road driving scores in age-adjusted analyses both for patients with glaucoma and for a control group (Lee et al., 2018).

Measures of *central* D_min_ have also been shown to be significantly associated with night-time driving performance assessed on a closed road. Significant associations between D_min_ and night-time pedestrian recognition distances were demonstrated in one study, and these were shown to be independent of driver age (*p* < 0.05) (Wood et al., 2014). In that study, drivers with impaired *central* D_min_ were significantly delayed in recognising pedestrians walking in place, resulting in a shorter recognition distance, which in the real world would make such a driver less able to stop in time to avoid a collision. In addition, in a closed road study of drivers with self-reported night-time driving difficulties, impaired D_min_ scores were significantly associated with poorer night-time driving scores in both the presence and the absence of intermittent glare (*p* = 0.001) (Kimlin et al., 2017).

Studies on the open road have also shown that impaired *central* D_min_ is significantly associated with poorer driving performance when assessed under in-traffic conditions, even when scored using standardised scales by blinded driving assessors. A larger on-road study of older drivers (Wood et al., 2008) found that D_min_ was a strong visual predictor of overall driving safety scores (*p* < 0.01) and also when combined with measures of cognitive and motor performance, predicting unsafe drivers with 91% sensitivity and 71% specificity. This finding was confirmed in two subsequent studies of older drivers (Anstey et al., 2020; Wood, Horswill et al., 2013a), which demonstrated the strong predictive capacity of a multi-domain battery that included assessment of D_min,_ for identifying unsafe older drivers. The significant association between D_min_ and driving performance has also been reported among older drivers with age-related macular degeneration, where D_min_ rather than visual acuity or contrast sensitivity was the only visual measure in age-adjusted analyses significantly associated with driving safety scores (*p* = 0.005) (Wood et al., 2018).

*Minimum drift rates* or *motion contrast thresholds* are typically measured using low-frequency drifting Gabor patches, represented as either the *minimum drift rate* at which grating direction can be identified or as the *minimum contrast* required to detect the direction of a Gabor drifting at a specific drift rate. Studies incorporating these measures have been shown to be significantly associated with a range of driving outcomes, including HPT response times, open road driving performance, and crash risk as described in the following sections.

Impaired *central* minimum drift rate using Gabor patches was significantly associated with delayed HPT times in two separate studies of drivers of a range of ages (Lacherez, Au et al., 2014a, Lacherez, Turner et al., 2014b), which remained significant even when corrected for age, visual acuity and contrast sensitivity. One of these studies also reported that minimum drift rate was significantly associated with the ability to identify deviations from normal lane-keeping, in videos of driving scenes filmed from a vehicle interior, which is related to optic flow (Lacherez, Turner, et al., 2014b). In a sample of older drivers with glaucoma and visually normal controls, impaired minimum drift rate was also associated with poorer HPT performance in age-adjusted analyses (*p* < 0.001) (Lee et al., 2018). A more recent study manipulated the minimum contrast required to detect the direction of a drifting Gabor, rather than the drift rate, and found that minimum contrast thresholds were not associated with HPT response times, whether the Gabors were presented centrally or peripherally (Sepulveda et al., 2023). These differences may arise because different characteristics of the Gabor were manipulated in different studies, with only those that measured drift rate finding associations between motion sensitivity and HPT response times (Lacherez, Au et al., 2014a, Lacherez, Turner et al., 2014b); however, this needs to be explored systematically in future studies.

Impaired *central* minimum drift rate has also been found to be associated with poorer on-road driving performance for older drivers aged ≥ 70 years with a range of eye conditions (Swain, McGwin, & Owsley, 2022). In that study, impaired minimum drift rate was defined by performance equal to or worse than the median value of the participant sample (≥ 0.14 Hz) and demonstrated that drivers with poorer motion sensitivity were more than twice as likely to have a worse standardised composite driving score (odds ratio (OR): 2.67; 95% confidence interval (CI): 1.14–6.26).

Drivers with impaired minimum drift rate (using the same definition of impairment) were also 2.6 times more likely to have had a state-reported MVC (in the previous 5 years) compared to those with better sensitivity of minimum drift rate in a larger sample of older drivers ≥ 70 years adjusted for age, visual acuity and contrast sensitivity (relative risk (RR) 2.6; 95% CI: 1.3–5.2) (Swain, McGwin, Wood, & Owsley (2021a). Similarly, at-fault prospective MVC and near-miss MVC risk recorded during naturalistic driving over a 6-month period was found to be significantly associated with impaired motion sensitivity using the same cut-point for impaired sensitivity of central drift rate in a sample of older adults (RR 1.9; 95% CI: 1.1–3.5) (Swain, McGwin, Wood et al., 2021b). Importantly, the technology used in this study, which included video footage, enabled MVCs to be defined as at-fault and near-miss MVCs to be identified, through assessment by independent raters who demonstrated high levels of inter-rater agreement.

Studies by Henderson and colleagues (2013, 2015) also explored the association between *peripheral* motion contrast thresholds (PMCTs) (for 0.4 c/deg drifting Gabor stimuli) presented at 15º eccentricity and driving simulator performance (STISIM driving simulator) in young and older drivers. In one study of young and older drivers, PMCTs were significantly correlated with rater scores of driving simulator performance, as well as simulator crashes (r = 0.63 and 0.66, respectively) for the older but not the young group (Henderson et al., 2013). This was supported by a study of young and older drivers where both the standard and an abbreviated version of the PMCT were shown to be significantly correlated with simulator crashes (r = 0.45–0.66), minimum distance of approach to all hazards (˗0.39 to ˗0.55), and lane deviation time (r = ˗0.48 to ˗0.64) in the older drivers only; interestingly the magnitude of correlations, shown in brackets, were not necessarily higher for the longer version of the PMCT (Henderson et al., 2015).

While motion perception has largely been assessed using 2D representations of motion stimuli, it has been suggested that measures of motion that use motion-in-depth (3D) cues might be more relevant to the driving task.

*Structure from motion* is a measure of the ability to derive structure from the perception of 3D structure-from-motion (SFM) assessed using computer-generated animation sequences, and has been shown to be associated with driving outcomes in a small series of studies of drivers with Parkinsons disease (PD) (Uc et al., 2006, Uc, Rizzo, Anderson et al., 2009a, Uc, Rizzo, Johnson et al., 2009b). Within a group of drivers with PD, reduced SFM was one of the strongest predictors of lane positioning (*p* < 0.01) and crash risk (OR 1.23; 95% CI: 1.02–1.49; *p* < 0.05) in a driving simulator assessed under low-contrast visibility conditions (Uc, Rizzo, Anderson, et al., 2009a). Declines in SFM, along with a range of other measures of visual function, were also significantly (*p* < 0.05) associated with increased on-road driving errors (such as errors in lane-keeping, turns, observing stop signs, speed control) in drivers with PD; however, only decreased distance visual acuity and UFOV total score were retained in the multivariate model (Uc, Rizzo, Johnson, et al., 2009b), while an earlier study found that reduced SFM was not associated with driving safety outcomes or visual search during on-road driving (Uc et al., 2006). While the findings for SFM are promising in this population, more studies are needed to further explore these effects including drivers with other types of impairment.

In a more recent study, the ability of 2D motion-defined letter (MDL) identification, 3D motion-in-depth sensitivity (MID), and dynamic visual acuity (DVA) to predict driving simulator performance was explored in visually normal, young participants (Wilkins et al., 2013). Findings suggest that MID, which involved judgement of which of two radially expanding flow fields of different velocities was the fastest, was most significantly associated with emergency braking (r = 0.46), while DVA was the strongest predictor of hazard detection (r = ˗0.61). Interestingly, a small subset of participants whose motion perception was trained weekly over a 6-week period demonstrated significant improvements in performance on all three motion tests and an associated reduction (improvement) of 0.17 s in braking reaction times compared to controls who did not undergo training. While these findings are promising, they have not been followed up and would need to be explored systematically in larger scale studies.

*Optic flow* is also likely to be relevant to driving in terms of judgement of a driver’s own speed, as well as that of other road users. Optic flow is also considered to be likely to contribute to time-to-collision decisions and gap judgements, as well as judgements of the direction of heading. Accordingly, the role of optic flow in driving has been explored in numerous studies whose experimental approach has typically been to manipulate the visual scenes in computer-based presentations or driving simulators to change available optic flow cues, rather than to explore the association between laboratory-based measures of optic flow and driving performance measures. Examples of driving tasks that have been explored in relation to optic flow include controlling speed and heading when entering a curve (Chen & Li, 2022; Köhler et al., 2023; Wilkie & Wann, 2003; Yotsutsuji & Kita, 2010), steering bends and changing lanes, (Okafuji et al., 2018; Xu & Wallis, 2020), judging whether it is safe to overtake and pass another vehicle (Gray & Regan, 2005), and responding to sudden braking of a lead vehicle (Lee, 1976).

Given the importance of optic flow information for these tasks, it is relevant to ask whether drivers with vision impairment (or higher-order visual processing difficulties) might differ in their capacity to perceive this optic flow information. While some research has suggested that older adults in particular may exhibit impairments in the perception of optic flow information (Guenot et al., 2023; Lich & Bremmer, 2014; Warren Jr et al., 1989), other research has also suggested that this form of motion may be relatively robust to visual declines related to ageing (Atchley & Andersen, 1998; Billino et al., 2008; Giguere et al., 2024).

### Summary and future directions

In summary, there is good evidence to suggest that psychophysical measures of visual motion sensitivity, including minimum displacement thresholds using RDKs and minimum drift rate or contrast thresholds using Gabor gratings, are associated with real-world driving outcome measures. The findings of the reviewed studies collectively suggest that motion sensitivity is important in the timely detection of hazards including pedestrians, as well as for visually guided vehicle control behaviours (e.g., lane-keeping).

There are, however, limitations in the evidence in this area, which has been largely restricted to exploration of the association between low-level, laboratory-based measures of motion sensitivity and driving performance and safety. Given the importance of understanding the role of motion sensitivity in driving, it is suggested that future research also incorporates measures of higher-level, global motion processing and motion segregation and discrimination to better assess the importance of these tasks in the driving environment. Studies specifically looking at biomotion cues or the systematic effects of illumination or visual degradation (e.g., fog or rain as visual noise) would also be insightful.

It is also important to recognise that the driving scene is highly complex and indeed difficult to quantify in an empirical way. Laboratory studies, or even closed-road studies, can at best hope to mimic only some aspects of the driving environment. It would therefore be useful to incorporate techniques such as naturalistic driving methodologies (studying participants’ driving behaviours in their own vehicles and environments using in-vehicle sensors during a period of everyday driving) to establish the extent to which laboratory measures of deficits in motion sensitivity are associated with driving performance and safety under in-traffic conditions.

Throughout this review, it has been noted that there are diverse measures of motion sensitivity, which index different perceptual outcomes. The extent to which impairments in any one of these domains reliably predict impairments in the other domains is not currently known. It would be useful to establish to what extent these measures relate to a common central ‘motion-processing’ deficit, and to what extent there may be unique deficits, and if the latter, whether these translate into specific driving challenges for affected individuals. One domain of motion sensitivity which might be of particular interest would be exploring how individual differences in ability to perceive optic flow information (e.g., with increasing age) impact on outcome measures of driving ability and safety. Finally, motion sensitivity is known to be affected by a variety of ocular conditions, as well as by physiological ageing. Better understanding the specific functions affected in individuals with ocular diseases could help to identify whether there are specific driving-related cues or driving behaviours that might be specifically challenging to individuals with these conditions, to better inform interventions or modifications which could be recommended for safer driving.

## Data Availability

Not applicable.
